# Synthesis and characterization of azo-guanidine based alcoholic media naked eye DNA sensor

**DOI:** 10.1098/rsos.160351

**Published:** 2016-11-02

**Authors:** Ataf Ali Altaf, Uzma Hashmat, Muhammad Yousaf, Bhajan Lal, Shafiq Ullah, Alvin A. Holder, Amin Badshah

**Affiliations:** 1 Department of Chemistry, University of Gujrat, Hafiz Hayat Campus, Gujrat 50700, Pakistan; 2 Department of Chemistry, Government College University, Faisalabad 38000, Pakistan; 3 Department of Energy Systems Engineering, Sukkur Institute of Business Administration, Sukkur, Pakistan; 4 Department of Chemistry, Quaid-i-Azam University, Islamabad 45320, Pakistan; 5 Department of Chemistry and Biochemistry, Old-Dominion University, Norfolk, USA

**Keywords:** DNA sensor, azo-guanidine, DNA binding constant, synthesis

## Abstract

DNA sensing always has an open meadow of curiosity for biotechnologists and other researchers. Recently, in this field, we have introduced an emerging class of molecules containing azo and guanidine functionalities. In this study, we have synthesized three new compounds (**UA1**, **UA6** and **UA7**) for potential application in DNA sensing in alcoholic medium. The synthesized materials were characterized by elemental analysis, FTIR, UV-visible, ^1^H NMR and ^13^C NMR spectroscopies. Their DNA sensing potential were investigated by UV-visible spectroscopy. The insight of interaction with DNA was further investigated by electrochemical (cyclic voltammetry) and hydrodynamic (viscosity) studies. The results showed that compounds have moderate DNA binding properties, with the binding constants range being 7.2 × 10^3^, 2.4 × 10^3^ and 0.2 × 10^3^ M^−1^, for **UA1**, **UA6** and **UA7**, respectively. Upon binding with DNA, there was a change in colour (a blue shift in the *λ*_max_ value) which was observable with a naked eye. These results indicated the potential of synthesized compounds as DNA sensors with detection limit 1.8, 5.8 and 4.0 ng µl^−1^ for **UA1**, **UA6** and **UA7**, respectively.

## 1. Introduction

DNA sensing is a vast field of interest in science. It is helpful to study the ways of inheritance in life. There are many chemical sensors used for DNA sensing which provide information about the composition of environment. There are a series of chemical and instrumental methods used for DNA sensing. Ethidium bromide (EtBr) is commonly used for DNA sensing. EtBr has the ability to show native fluorescence, but it shows 20–25 times more fluorescence on intercalation with DNA double helix [1–3]. On the basis of this significant fluorescence, EtBr shows its popularity in the field of science and is used extensively in molecular biology laboratories [3,4]. It is used to detect DNA as well as RNA. EtBr may act as a carcinogen as it affects mitochondrial DNA of proliferation cells and inhibits its replication. It can also act as a teratogen as it adversely affects the human reproductive system and disturbs the development of an embryo. EtBr can also act as a mutagen as it has the ability to intercalate with DNA and cause deformation, which adversely impacts DNA replication and transcription. The effectiveness of EtBr depends upon its exposure to an organism and the circumstances [5–8]. These adverse effects of EtBr develop interest for the search of alternatives to avoid the use of EtBr. So, some alternatives have been reported which are less dangerous and have good performance, for example SYBR, TOTO, YOYO families of dye [9–11]. SYBR Green-I was considered the best alternative of EtBr in the sense of safety [1], but a few years ago, it was discovered that SYBR Green-I is more mutagenic than EtBr because it binds more strongly with DNA and causes DNA deformation. So far these studies lead to the development of a hypothesis, i.e. the dyes with weak DNA binding ability and better probing ability would be the best DNA sensing agents [12].

In our most recent study, we synthesized novel azo-guanidine derivatives. These synthetic compounds were characterized by instrumental techniques such as elemental analysis, FTIR spectroscopy, NMR spectroscopy, UV-visible spectroscopy and cyclic voltammetry. Their DNA detection ability, binding behaviour and binding constants have been discussed in this manuscript.

## 2. Experimental: material and methods

Dithizone, mercuric chloride (HgCl_2_), triethylamine (Et_3_N), 2-nitroaniline, 3-nitroaniline and 4-nitroaniline were purchased from Aldrich. DNA (deoxyribonucleic acid) was purchased from Acros Organics. Solvents such as chloroform, DMF and ethanol were purchased from Aldrich, and petroleum ether and ethyl acetate were purchased from Fischer Scientifics; all these solvents were purified before use according to the reported protocol [13]. Melting points were measured on BIO COTE Model SMP10 melting apparatus. Elemental composition was estimated on Thermo Scientific Flash 2000 organic elemental analyser. FTIR was recorded on Thermo Scientific Nicolet-6700 FTIR spectrophotometer. NMR spectrum was recorded on BRUKER AVANCE 300 MHz NMR spectrometer and UV-visible spectrum was recorded on UV-visible spectrophotometer Shimadzu 1800.

### 2.1. Synthesis of 1-(phenylamino)-2-(3-nitrophenyl)-3-(phenylimino)guanidine (**UA1**)

Dithizone (1 g, 3.9 mmol) and HgCl_2_ (1.06 g, 3.9 mmol) were added to a two neck round bottom flask containing 30 ml of anhydrous DMF. The reaction flask was fitted with magnetic stirrer and heating plate under nitrogen atmosphere. 3-Nitroaniline (0.6 g, 3.9 mmol) and triethylamine (1.08 ml, 7.8 mmol) were added to the reaction mixture after 30 min of stirring at room temperature. The progress of the reaction was monitored on TLC using the petroleum ether and ethyl acetate (9 : 4) solvent system. The reaction was completed with the formation of black precipitate (HgS) in the container. The reaction mixture was filtered with sintered glass crucible. The crude product was obtained from the filtrate by the evaporation of the solvent and was washed with excess water. Purple coloured **UA1** was re-crystallized using CHCl_3_. Yield 64% (0.899 g, 2.5 mmol), m.p. decompose at 210°C. Elemental analysis for C_19_H_16_N_6_O_2_ calc. C, 63.33; H, 4.44; N, 23.33; found C, 63.28; H, 4.45; N, 23.36%. UV-visible (*λ*_max_, nm) 265, 490; FTIR (*υ*, cm^−1^) 3745, 3624, 3218, 3019, 2926, 1690, 1525, 1484, 1460, 1339, 1228, 1140, 1074, 994, 918, 832, 758, 684, 596, 519, 459, 434; ^1^H NMR (400 MHz, CDCl_3_) *δ* 8.30–8.10 (2H, m), 7.84 –7.72 (3H, m), 7.54 (1H, t, *J* = 6.0 Hz), 7.37–7.31 (3H, m), 7.17 (2H, t, *J* = 6.0 Hz), 6.79–6.60 (3H, m), 3.72 (1H, s), 2.94 (1H, s); ^13^C NMR (100 MHz, CDCl_3_) *δ* 163.8, 153.3, 151.5, 150.5, 148.8, 131.7, 130.1, 129.3, 128.8, 126.5, 122.3, 119.4, 115.7, 114.0, 113.1.

### 2.2. Synthesis of 1-(phenylamino)-2-(2-nitrophenyl)-3-(phenylimino)guanidine (**UA6**)

Compound **UA6** was synthesized in the same way as **UA1,** using 2-nitroaniline in place of 3-nitroaniline. Yield is 64% (0.899 g, 2.5 mmol), m.p. 150°C. Elemental analysis for C_19_H_16_N_6_O_2_ is calc. C, 63.34; H, 4.45; N, 23.34; found C, 63.36; H, 4.41; N, 23.36. UV-visible (*λ*_max_, nm) 258, 524; ^1^H NMR (400 MHz, CDCl3) *δ* 8.20–7.80 (3H, m), 7.60–7.29 (6H, m), 7.16 (2H, t, *J* = 6.0 Hz), 6.80–6.60 (3H, m), 4.69 (1H, s), 2.96 (1H, s); ^13^C NMR (100 MHz, CDCl3) *δ* 163.3, 161.4, 159.3, 153.3, 149.5, 149.4, 148.8, 130.0, 129.9, 129.9, 129.3, 128.8, 122.3, 119.4, 119.4, 119.4, 113.1, 111.2, 110.9, 108.6, 108.4.

### 2.3. Synthesis of 1-(phenylamino)-2-(4-nitrophenyl)-3-(phenylimino)guanidine (**UA7**)

Compound **UA7** was synthesized in the same fashion as for **UA1,** using 4-nitroaniline in place of 3-nitroaniline. Yield is 64% (0.899 g, 2.5 mmol). Elemental analysis for C_19_H_16_N_6_O_2_ is calc. C, 63.34; H, 4.45; N, 23.34; found C, 63.36; H, 4.41; N, 23.36. UV-visible (*λ*_max_, nm) 280, 517. ^1^H NMR (400 MHz, CDCl3) *δ* 8.30–8.20 (4H, m), 8.07 (1H, s), 7.62–7.45 (6H, m), 7.20–7.10 (2H, m), 6.78–6.69 (2H, m), 3.00 (s). ^13^C NMR (100 MHz, CDCl3) *δ* 163.7, 154.7, 152.4, 148.8, 139.6, 130.0, 129.3, 128.8, 125.9, 122.3, 121.2, 119.4, 113.1.

### 2.4. DNA binding studies

Sodium salt degraded DNA (Salmon Testes) was purchased from Acros organics. The small amount of sodium salt of DNA was dissolved in distilled water and its concentration was measured by spectrophotometer, which gave 6600 M^−1^ cm^−1^ molar absorption coefficient at 260 nm. The purity of DNA was measured by the appearance of absorption peaks at 260 and 280 nm [14,15]. The solution was buffered at pH 7 by using phosphate buffer (0.051 g of NaH_2_PO_4_ and 0.169 g of Na_2_HPO_4_ dissolved in 100 ml distilled water). The prepared DNA solution was stored below 4°C and should be used within 4 days.

### 2.5. UV-visible spectroscopy

UV-visible spectrum was obtained by using UV/visible spectrophotometer Shimadzu 1800. The interaction of compound **UA1** with DNA was carried out by using aqueous ethanol (containing 20% water and 80% ethanol) and phosphate buffer of pH 7. The spectrum of compound **UA1** was recorded in the absence and presence of varying concentration of DNA [16].

### 2.6. Viscometry

The viscometry experiments were performed on Ostwald viscometer at constant temperature 25 ± 1°C. The flow time was recorded with a digital stopwatch. For viscometry, a 100 µM solution of DNA was prepared in 80% ethanol. The solution was buffered at pH 7 by using 10 mM phosphate buffer. The time of flow was recorded for the solution in the absence and presence of 20, 40, 60, 80, 100 and 120 µM concentration of test sample [17].

### 2.7. Cyclic voltammetry

For cyclic voltammetry analysis, the compound **UA1** was analysed by using glassy carbon electrode in reference with standard calomel electrode (SCE) in aqueous ethanol containing 20% of water and 80% of ethanol. Electrolyte used for the CV is TBAP (tetrabutyl ammonium phosphate) of 1 × 10^−3^ M. The anodic peak was observed in the range of −1.6 to −0.2 V at a scanning rate of 100 mV s^−1^. The cyclic voltammetry of compound **UA1** was studied by varying the concentration of DNA at pH 7 and the electrodes were cleaned before every experiment [18,19].

## 3. Results and discussion

### 3.1. Synthesis and characterization

**Scheme 1.**
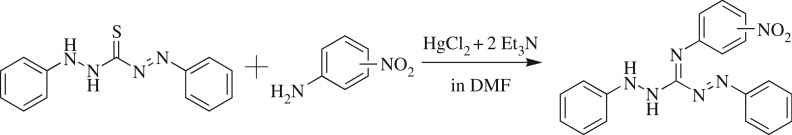
Reaction for the synthesis of test molecules: **UA1** (nitro group is at *meta* position), **UA6** (nitro group is at *ortho* position) and **UA7** (nitro group is at *para* position).

The compounds **UA1**, **UA6** and **UA7** were synthesized by the guanylation reaction between dithizone and nitro-anilines by using Hg^2+^ as a sulfur abstracting agent (scheme 1). The guanylation reaction is well reported for the synthesis of such guanidine derivatives [20,21].

These novel compounds have been structurally characterized by instrumental methods like elemental analysis, different spectroscopic techniques and cyclic voltammetry. The elemental analysis shows that the suggested molecular composition is in good agreement with the experimental results and also confines the bulk purity of the compounds. The vibrational spectroscopy shows the appearance of C = N stretching band at 1510–1527 cm^−1^ indicating the formation guanidine nucleus in accordance with the literature data [21,22]. The remaining FTIR data as reported in the experimental section explain the structural features of the respective compounds.

**Figure 1.**
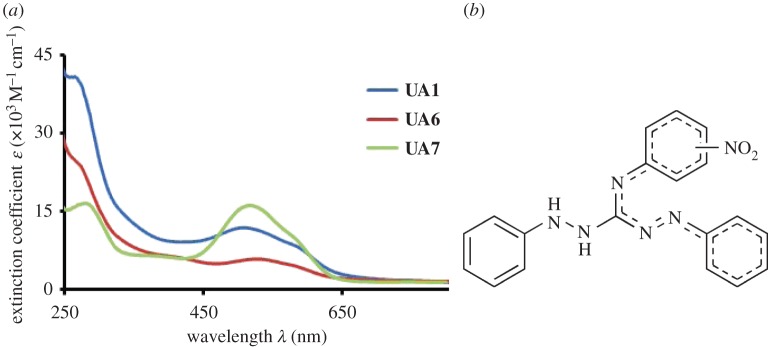
(*a*) The UV-visible spectra of synthesized compounds taken for 50 µM solution of each compound in 80% ethanol solution (*b*). The extended resonance in the general structure of the compounds.

In solution phase, the synthesized molecules have been characterized by NMR (^1^H, ^13^C), UV-visible spectroscopy and cyclic voltammetry. The NMR data were recorded in CDCl_3_ and TMS was referenced as the internal standard. Using the ^1^H NMR, the successful formation of **UA1**, **UA6** and **UA7** can be judged from the proton chemical shift of the NH groups; those appeared in the range of 2.94–4.69 ppm and the total number of protons (integration value) appeared in the spectra matches with the suggested structures. In ^13^C NMR, the synthesis of **UA1**, **UA6** and **UA7** can be confirmed by the presence of the most downfield guanidine carbon at 163.8, 163.3 and 163.7 ppm, respectively. In addition, the number of carbon atoms that appeared in ^13^C NMR spectra for these compounds is in agreement with their confined structures. The UV-visible spectra of all the synthesized compounds were taken in 80% ethanol/water (20%) solution. The UV-visible spectra for all the synthesized compounds have been presented in figure 1. The characteristic UV-visible spectrum contains two bands. One band is in the UV region of the spectrum at 250–320 nm ranges with *λ*_max_ at 265, 258 and 280 nm for **UA1**, **UA6** and **UA7**, respectively. This UV-region band can be assigned to the phenyl ring based π–π* electronic transitions in these compounds. The other band in these compounds appeared in the visible region of the spectrum with a lower energy shoulder. The bands appeared with *λ*_max_ at 490,524 and 517 nm for **UA1**, **UA6** and **UA7**, respectively, and can be assigned to π–π* transition of the azo-group based on the extended resonance system as shown in figure 1. The shoulders appeared at about 580 nm for all compounds and can be assigned to *n*–π* transition of the system.

**Figure 2.**
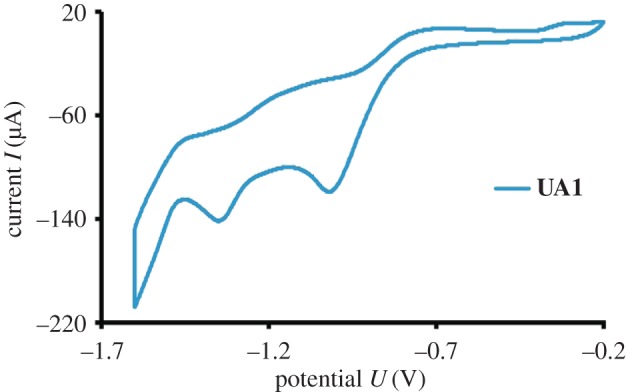
Cyclic voltammogram of 50 µM **UA1** in 80% ethanol, 0.1 M TBAP was used as the supporting electrolyte.

The compound **UA1** was also characterized electrochemically by cyclic voltammetry in 80% ethanol. TBAP was used as the supporting electrolyte as described in the Experimental section. The cyclic voltammogram has been presented in figure 2. Two peaks were observed in the voltammogram of **UA1** at potential values of −1.019 and −1.35 V, at current values of −119 and −142 µA, respectively. These bands can be assigned to the electrochemical reduction of the nitro and azo groups, respectively, present in **UA1** as described in the literature [23–25].

### 3.2. DNA binding studies

**Figure 3.**
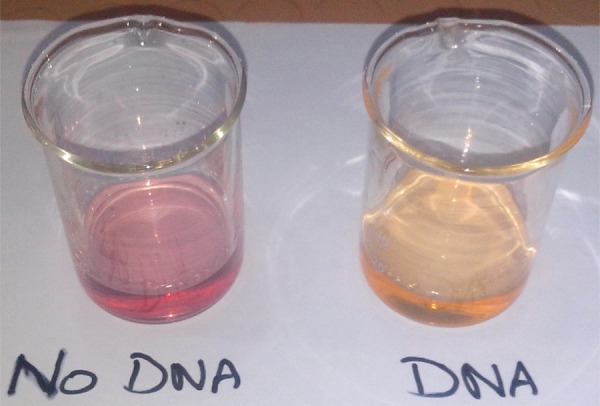
The change in the colour of the ethanolic solution of test sample **UA6** after its interaction with DNA.

DNA binding study is very important to evaluate the DNA detection ability of a material. The synthesized compounds interact with DNA and show a clear colour change for the reaction as shown in figure 3. To understand the colour change of solution, the reaction between samples and DNA has been probed by using different techniques. In this regard, the DNA interaction of the synthesized samples has been studied by UV-visible spectroscopy and viscosity measurement.

### 3.3. UV-visible spectrometric titration

**Figure 4.**
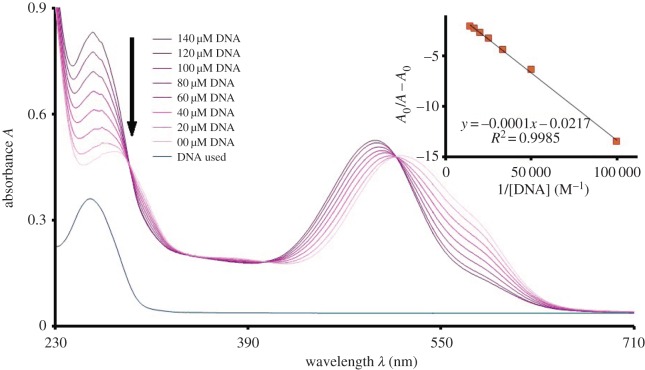
UV-visible absorption spectrum of **UA7** (50 µM) without DNA and with DNA (20–140 µM) in 80% ethanol, buffer (0.1 M, pH = 7.0). Inset is the plot between *A*_0_/(*A* − *A*_0_) and 1/[DNA] for the calculation of binding constant.

UV-visible spectroscopy is a very effective technique to study DNA interaction. It provides very clear clues about the interaction of small molecules with DNA. The electronic energy changes during the reaction between DNA and the interacting molecules. The electronic spectra of all the synthesized compounds have been explained in the previous section. In the case of **UA7**, the addition of DNA causes a rapid change in the UV-visible spectrum of the sample as shown in figure 4. After the addition of DNA, three types of changes in the spectra of **UA7** were seen i.e. increase in absorbance at 260 nm and around 500 nm, blue shift in *λ*_max_ from 517 to 495 nm and decrease in absorbance around 580 nm. The increase in absorbance seen around 260 nm region is due to the increase in the concentration of DNA, as it is the specific region for DNA bases [26]. The hyperchromism around 500 nm indicates the increasing probability of electrons in the π–π* transition of the extended resonance system (indicated in figure 1). From this observation, it can be commented confidently that electron-rich DNA donates the electrons to the π-orbitals of the system. The hypsochromic shift of 22 nm also supports the statement, as increasing electron density in π-orbitals stabilizes the orbitals, which causes an increase in the energy gap between π and π* orbitals.

**Figure 5.**
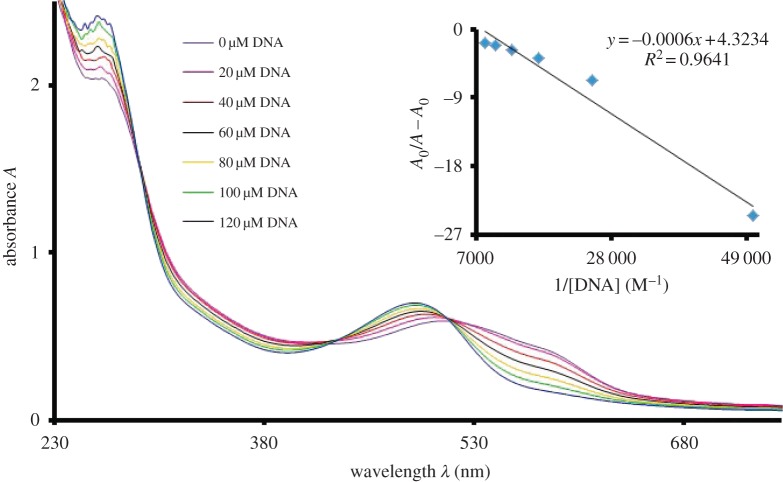
UV-visible spectrum of **UA1** (50 µM) without DNA and with DNA interaction (20–140 µM) in 80% ethanol, phosphate buffer (0.1 M, pH = 7.0). Inset is the graph between *A*_0_/(*A* − *A*_0_) and 1/[DNA] for the calculation of binding constant.

**Figure 6.**
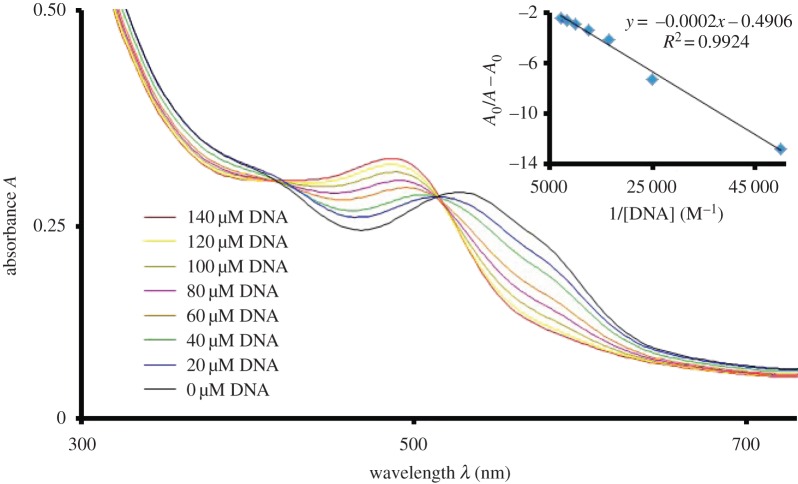
UV-visible spectrum of compound of **UA6** (50 µM) without DNA and with DNA interaction (20–140 µM) in 80% ethanol, phosphate buffer (0.1 M, pH = 7.0). Inset is the graph between *A*_0_/(*A* − *A*_0_) and 1/[DNA] for the calculation of binding constant. The full scan from 200 to 800 nm for the experiment is provided as electronic supplementary material, figure S1.

Hypochromism around 580 nm indicates the reduced n–π* electronic transition. This may be due to the involvement of a non-bonded electron in H-bonding between **UA7** and the polar hydrogens of DNA. This H-bonding further supports the penetration of **UA7** into the DNA helical structure, which enhances the chances of π-stacking. As a result of stacking, the interaction between π-orbitals and DNA increases, which also support the blue shift for π–π* transition. The existence of hyperchomism and hypochromism results in the formation of an isosbestic point, which confers the presence of two types of interactions (H-bonding and π-stacking) in equilibrium. Compounds **UA1** and **UA6** behave in similar ways for UV-visible spectroscopic analysis as a result of their interaction with DNA. As shown in figures 5 and 6, there exist isosbestic points in the UV-visible spectroscopic response, upon the variation of DNA concentration, of **UA1** and **UA6**. There is hypsochromic shift at the visible region *λ*_max_ of both the compounds. The quantitative blue shift of all the compounds has been described in table 1 along with the other data.

**Table 1.** Comparative data for synthesized compounds and some reported DNA staining agents. CW = current work. The detection limit of the synthesized compounds is calculated by change in absorbance (Δ*A* = 0.01) of sample, at *λ*_max_ and using Lambert-Beer's law, for the changing concentration of DNA [27,28].

s. no.	compound	mode of interaction	binding constant *K* (M^−1^)	change in *λ*_max_ (nm)	detection limit (ng µl^−1^)	references
1	**UA1**	electrostatic	7.2 × 10^3^	24	1.8	CW
2	**UA6**	electrostatic	2.4 × 10^3^	42	5.8	CW
3	**UA7**	electrostatic	0.2 × 10^3^	22	4.0	CW
4	AG	electrostatic	10^4^	10	15	[3]
5	EtB	intercalation	>10^5^	44	1.0	[29,30]
6	SYBR G-I	groove binding	>10^6^	27	0.06	[4,31]
7	TOTO-I	intercalation	10^9a^	—	0.02	[32]
8	YOYO-I	intercalation	>10^10^	—	0.5	[33–35]
9	methylene blue	intercalation/groove binding	>10^4^	3	5.0	[36,37]

Based upon the change in the concentration of DNA, at the constant concentration of test sample, the DNA binding constant has been calculated. The following host--guest equation (3.1) was used to calculate the DNA binding constant (*K*).

3.1 $$\frac{A_{0}}{A-A_{0}}=\frac{\varepsilon_{G}}{\varepsilon_{H-G}-\varepsilon_{G}}+\frac{\varepsilon_{G}}{\varepsilon_{H-G}-\varepsilon_{G}}\times \frac{1}{K[\mathrm{DNA}]},$$

where *A*_0_ and *A* are the absorbance of test sample, at *λ*_max_, in the absence and presence of DNA respectively. Similarly ε_G_ and ε_H−G_ are the molar absorptivities of test sample, at *λ*_max_, in the absence and presence of DNA, respectively. The value of binding constant (*K*) was calculated from the intercept to slope ratio of the linear plot between *A*_0_/(*A* − *A*_0_) and 1/[DNA]. Further, from the binding constant (*K*), the change in Gibb's free energies (Δ*G*) of the reactions has been evaluated using the relation Δ*G* = −*RT* ln (*K*), where *R* is the gas constant and *T* is the absolute temperature. The binding constant and Gibb's free energies data for all the samples has been described in table 1 with other data. The negative values of Δ*G* indicate the spontaneous reaction between the test samples and DNA.

### 3.4. Viscometric titration

**Figure 7.**
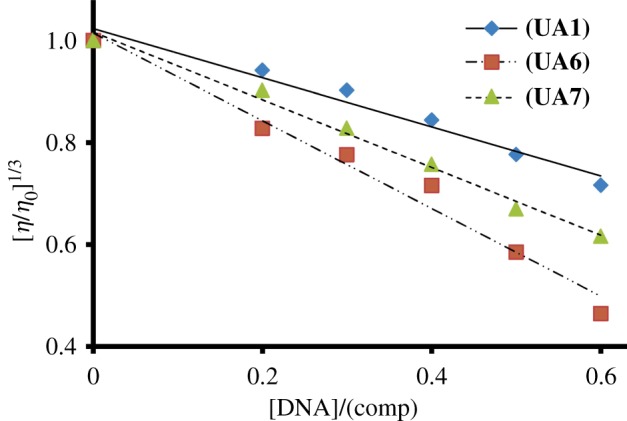
Variation of relative viscosity [*η*/*η*_o_]^1/3^ of 0.1 mmol l^−1^ DNA with increasing concentration of **UA1**, **UA6** and **UA7**.

Optical studies alone are insufficient to conclude the binding mode of small molecules with DNA. To understand the binding mode, hydrodynamics studies are considered to be the least ambiguous [17]. Viscosity measurement is normally used as a hydrodynamic tool for these kinds of studies. Generally, two types of binding modes are associated with the change in viscosity of DNA solution. These modes are intercalation and electrostatic interaction. The intercalation of small molecules into the DNA double helical structure increases its hydrodynamic radius and hence increases the viscosity of the solution [38]. On the other hand, the electrostatic interaction leads to formation of agglomerates which reduce the number of independent moving particles in the solution, resulting in the decrease of viscosity of the solution [39]. In this study, all the samples **UA1**, **UA6** and **UA7** were evaluated with viscosity measurement to understand their mode of interaction with DNA. The result obtained has been presented in figure 7 as a plot of relative viscosity versus concentration of the small molecules. The graph in figure 7 shows that the increasing concentration of test samples (**UA1**, **UA6** and **UA7**) cause the decrease in relative viscosity of the DNA solution. The decrease in viscosity clearly indicates the electrostatic-type interaction between test samples (**UA1**, **UA6** and **UA7**) and DNA.

### 3.5. Cyclic voltammetric titration

**Figure 8.**
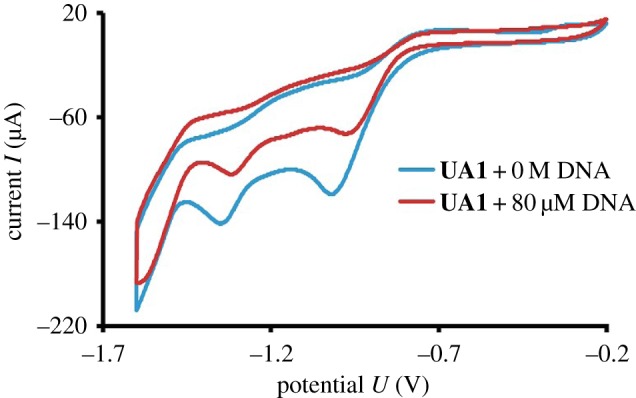
Cyclic voltammogram of 50 µM **UA1** without DNA and with 80 µM DNA.

To further confirm the mode interaction between the synthesized compounds and DNA, electrochemical probing of the reaction between **UA1** and DNA was performed by cyclic voltammetry. Cyclic voltammetry is a fine tool to understand the interaction between small molecules and DNA [40]. It provides a lot of information about such reactions like: type of interaction, binding site, size of binding site, diffusion coefficient and the binding constant [3]. In this study, **UA1** was examined by cyclic voltammetry in the presence and absence of DNA. The CV behaviour of **UA1** has been discussed in the previous characterization section. There were two peaks observed in voltammogram of free **UA1** at potential values of −1.019 and −1.35 V with current values of −119 and −142 µA, respectively. As shown in figure 8, on the addition of 80 µM DNA in **UA1** solution, the peak potential shifted towards the positive side (anodic side) by 39 and 32 mV, respectively.

The peak current was decreased by 32 and 26% in magnitude. This decrease in current peaks showed the slow formation **UA1**–DNA complex and by forming complex, the concentration of free compound **UA1** decreased. This decreasing of free compound concentration is responsible for lowering current values in cyclic voltammogram and showed DNA binding to facilitate the reduction of **UA1**. The shifting in peak potential suggests the nature of interaction between small molecules and DNA [40]. In the current experiment, peak potential was shifted towards the anodic side after the addition of 80 µM DNA which explained electrostatic mode of interaction, i.e. hydrogen bonding between DNA and **UA1**. This anodic shift also showed that the complex is formed, which enhances the reduction of **UA1** under the electron-rich environment of DNA.

**Figure 9.**
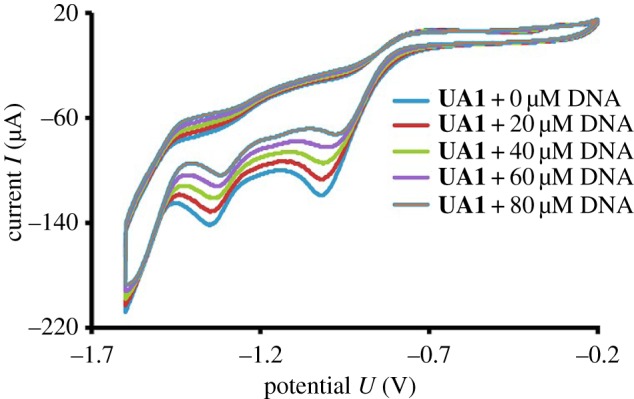
Cyclic voltammogram of 50 µM **UA1** without DNA and with increasing concentration of DNA.

Based upon the change in peak current and DNA concentration (as shown in figure 9), the binding constant (*K*) and size of binding site (*S*) were calculated according to literature methods [3]. The following equation (3.2) was used for the calculation of *K*_b_ [19].

**Figure 10.**
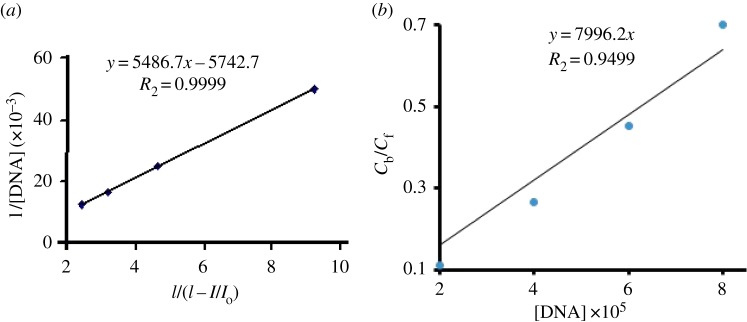
(*a*) The plot of 1/[DNA] (M^−1^) versus 1/(1 − *I*/*I*_o_) (unitless quantity) for the calculation of binding constant *K*_b_. (*b*) The graph between *C*_b_/*C*_f_ (unitless quantity) and [DNA] (M) for the calculation of binding site size *S*.

3.2 $$\frac{1}{\left[\text{DNA}\right]}=\frac{K_{b}(1-A)}{\left(1-I/I_{o}\right)}-K_{b},$$

where *I*_o_ and *I* are the peak current of **UA1** solution in the absence and presence of DNA, respectively; *A* is proportionality constant. The plot of 1/[DNA] versus 1/(1 − *I*/*I*_o_) gave a straight line (figure 10*a*) with *y*-intercept equal to the binding constant (*K*_b_ = 5.74 × 10^3^ M^−1^). In this way, the calculated value is in agreement with that observed by UV-visible spectroscopy. The binding site size (*S*) is calculated in accordance with the following literature reported equation (3.3) [3].

3.3 $$\frac{C_{b}}{C_{f}}=\frac{K_{b}[\text{DNA}]}{2S},$$

where *C*_f_ and *C*_b_ symbolize the concentration of the free and DNA-bound species, respectively. The *C*_b_/*C*_f_ ratio was determined as *C*_b_/*C*_f_ = (*I*_o_ − *I*)/*I*, and the plot of *C*_b_/*C*_f_ versus [DNA] (figure 10*b*) yields the slope *K*_b_/2*S* [3]. *S* is calculated as 0.36 by using the value of *K*_b_ from (3.2). The small value of *S* also confirms the electrostatic interaction between **UA1** and DNA.

In this article, only **UA1** was studied using cyclic voltammetry and the remaining two were assumed to behave similarly, as all the compounds showed similar kinds of observations in other studied techniques.

### 3.6. DNA sensor properties

All of the tools used for the estimation of DNA binding mode confer the electrostatic interaction between the test samples and DNA. UV-visible spectroscopic studies infer the clear colour change and fine change in the *λ*_max_ of the test samples (**UA1**, **UA6** and **UA7**) upon interaction with DNA. As hypothesized in the introduction, for a compound to be a good sensor for DNA, it is required to bind weakly with DNA and present better change in probing property. The data in table 1 indicate that the sensing materials already in use either interact strongly with DNA or intercalate into the DNA helical structure [29–35]. The intercalating compounds normally cause mutation [41,42]. Data also show that the in-use materials give less *λ*_max_ shifting in comparison to the compounds synthesized in this study.

**Figure 11.**
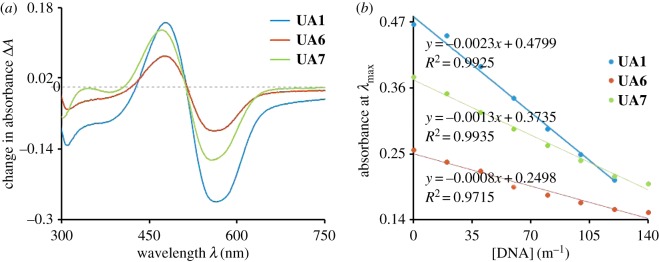
(*a*) Plot of differential absorbance (Δ*A*) versus wavelength, where Δ*A* is the change in absorbance of the test sample after the addition of 120 µM DNA to sample solution. (*b*) The graph between absorbance (Abs) and [DNA] for the calculation of detection limit.

Among the tested samples, in this study, **UA6** shows better change in *λ*_max_ in comparison with the others as shown in figure 11. However, the detection limit of the synthesized compounds is comparable to the materials already in use.

### 3.7. Conclusion

Three potential DNA sensors have been successfully synthesized and structurally characterized in the solid and solution phase. Their DNA binding potency is estimated with a variety of techniques. It is concluded that the binding mode is electrostatic and binding strength is moderate and is less than the other existing DNA sensing molecules. The good changes in *λ*_max_ conclude that these molecules may be the better sensors for DNA. Also, these molecules are good to use in ethanol whereas the existing molecules cannot be used in alcoholic media. The super-cooled ethanol is used for the quenching of DNA from water solution during the extraction process. During quenching, some of the DNA becomes soluble in ethanol. Therefore, the estimation of DNA is important in alcoholic media for total quantification.

## Supplementary Material

Ataf Ali - Excell Data

## Supplementary Material

Supplementary material from “Synthesis and characterization of azo-guanidine based alcoholic media naked eye DNA sensor”

## Data accessibility

All the datasets supporting this article are available at the Dryad Digital Repository: http://dx.doi.org/10.5061/dryad.fs001 [43].
