# Supplementary figures and images for: Synthesis and characterization of azo-guanidine based alcoholic media naked eye DNA sensor
Source: R Soc Open Sci. 2016 Nov 2;3(11):160351. doi: 10.1098/rsos.160351 (PMC5180111; doi:10.1098/rsos.160351)

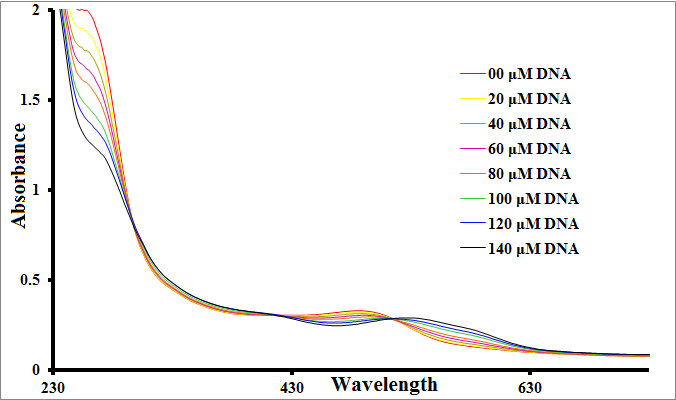

Supplement: Supplementary material from “Synthesis and characterization of azo-guanidine based alcoholic media naked eye DNA sensor” [file rsos160351supp2.png]
